# Comparative Evaluation of Ultrasound-Assisted Extraction and Hydrodynamic Cavitation Under Optimized Solvent Conditions for Phenolic Recovery from Lemon By-Products

**DOI:** 10.3390/foods15081418

**Published:** 2026-04-18

**Authors:** Gabriele Ballistreri, Ignazio Maria Gugino, Martina Papa, Michele Canale

**Affiliations:** Council for Agricultural Research and Economics (CREA), Research Center for Olive, Fruit and Citrus Crops, Corso Savoia 190, 95024 Acireale, Italy; ignaziomaria.gugino@crea.gov.it (I.M.G.); martina.papa@crea.gov.it (M.P.); michele.canale@crea.gov.it (M.C.)

**Keywords:** lemon by-products, phenolic compounds, solvent optimization, ultrasound-assisted extraction, hydrodynamic cavitation, comparative extraction, circular bioeconomy

## Abstract

Efficient recovery of phenolic compounds from citrus processing by-products requires optimized solvent systems and reliable frameworks for comparing emerging extraction technologies. In this study, a solvent system was first optimized to maximize phenolic recovery from lemon (*Citrus limon* (L.) Burm. f.) processing by-products, enabling a standardized comparison of ultrasound-assisted extraction (UAE) and hydrodynamic cavitation (HC). A preliminary solid–liquid extraction screening using different water:ethanol ratios (*v*/*v*) identified a 50:50 hydroalcoholic mixture as the optimal solvent system for recovering phenolic compounds. HPLC analysis confirmed the presence of major flavanones (eriocitrin and hesperidin) and hydroxycinnamic acids (caffeic, *p*-coumaric, sinapic, and ferulic acids). Antioxidant capacity was assessed using complementary assays (Folin–Ciocalteu, DPPH, and ORAC) to provide a comprehensive evaluation of antioxidant activity. Under optimized solvent conditions, UAE significantly improved the recovery of total flavanones (+25.9%), hydroxycinnamic acids (+10.3%), total polyphenols (+20.5%), DPPH activity (+6.0%), and ORAC values (+9.6%) compared with conventional extraction. HC further enhanced extraction performance, increasing flavanone recovery by 12.0%, hydroxycinnamic acids by 7.2%, total polyphenols by 5.2%, and antioxidant activity (DPPH and ORAC) by 11.4% and 2.0%, respectively, relative to UAE. Following ethanol removal and concentration, HC-derived extracts showed the highest phenolic content and antioxidant capacity. These results demonstrate that solvent optimization, combined with a standardized comparison of extraction technologies, enhances phenolic recovery from lemon processing by-products. The findings indicate that HC is a promising, scalable approach for the sustainable recovery of bioactive compounds from citrus side-streams. The novelty of this work lies in the integration of solvent optimization with a systematic and standardized comparison of UAE and HC, providing a reproducible framework for evaluating emerging extraction technologies and highlighting the enhanced performance and scalability potential of HC for phenolic recovery from citrus processing by-products.

## 1. Introduction

In recent years, increasing attention has been devoted to the identification and characterization of plant secondary metabolites due to their potential applications in human health and industrial sectors [1,2]. These compounds, including phenolics, flavonoids, and other bioactive molecules, are widely recognized for their antioxidant and anti-inflammatory properties and for their distribution across different plant tissues.

The recovery of phenolic compounds from citrus processing by-products has attracted increasing interest due to the high concentration of bioactive molecules present in these matrices. Lemon (*Citrus limon* (L.) Burm. f.) by-products, including peel, pulp, and seeds, are mainly generated by the citrus juice and essential oil processing industries, as well as by other industrial applications such as citrus-based food products, and represent an abundant agro-industrial side-stream. It has been reported that approximately 33% of global citrus production is industrially processed, generating large volumes of by-products that may account for around 50% of the processed fruit mass [3]. These by-products are rich in flavonoids and phenolic acids with well-documented antioxidant and health-promoting properties [3,4,5]. The efficient recovery of these compounds is therefore of growing relevance for the development of functional ingredients for food, nutraceutical, and cosmetic applications, while simultaneously contributing to the more sustainable management of food processing side-streams within circular bioeconomy strategies [6,7].

The extraction of phenolic compounds from citrus by-products has been widely investigated using both conventional and emerging technologies. Traditional solid–liquid extraction methods, such as maceration with organic solvents, remain commonly employed but are often limited by long processing times, high solvent consumption, low selectivity, and environmental concerns. To address these limitations, several green extraction technologies have been explored in recent years, including ultrasound-assisted extraction (UAE) [8], microwave-assisted extraction [9], pressurized liquid extraction [10], enzyme-assisted extraction [11], and the use of natural deep eutectic solvents (NaDES) [12]. These approaches can enhance extraction efficiency while reducing solvent consumption, processing time, and thermal degradation of sensitive compounds.

Among these technologies, UAE is one of the most frequently applied techniques for the recovery of phenolic compounds from lemon by-products due to its operational simplicity and effective enhancement of mass transfer through acoustic cavitation [13,14]. However, UAE is predominantly implemented at the laboratory scale and may present limitations when translated to larger processing volumes. Hydrodynamic cavitation (HC), in contrast, is an emerging non-thermal technology that generates cavitation phenomena through pressure fluctuations in liquid flow, leading to intense turbulence, bubble collapse, and enhanced disruption of plant tissues. This process can accelerate mass transfer and facilitate the release of matrix-bound phenolics from complex agro-industrial substrates [15,16,17,18,19,20,21]. Moreover, HC has been increasingly recognized for its scalability and suitability for continuous-flow processing systems, making it particularly attractive for industrial applications within circular bioeconomy frameworks [22].

Despite the growing interest in these extraction technologies, direct comparisons between UAE and HC applied specifically to lemon processing by-products remain limited. In particular, few studies have evaluated these technologies under strictly comparable experimental conditions, making it difficult to accurately assess their relative extraction efficiency and technological potential. One critical factor influencing extraction performance is solvent composition. Hydroalcoholic mixtures, typically based on water and ethanol, are widely used for the recovery of phenolic compounds due to their food-grade status and their ability to balance solvent polarity and matrix permeability. However, differences in solvent systems can strongly influence extraction yields, potentially confounding comparisons between extraction technologies.

For this reason, establishing an optimized solvent system prior to technological comparison represents an important step toward a standardized evaluation of extraction performance. In citrus matrices, phenolic compounds such as flavanones and hydroxycinnamic acids may occur both in free form and associated with the plant matrix, requiring appropriate solvent polarity and extraction conditions to ensure efficient recovery.

Based on these considerations, the present study investigated how solvent composition interacts with extraction technology to influence phenolic recovery and antioxidant performance from lemon processing by-products. A preliminary solvent screening was conducted to identify the optimal water–ethanol ratio for phenolic extraction. Subsequently, UAE and HC were applied under comparable conditions to evaluate their relative performance.

Previous studies have shown that UAE enhances phenolic recovery primarily through acoustic cavitation, which improves mass transfer and promotes cell disruption, although limitations related to energy distribution and scalability have been reported [13,14]. In contrast, HC has been described as a potentially more efficient and scalable technique due to the generation of more uniform cavitation conditions and its compatibility with continuous-flow processing, leading to improved extraction performance in plant matrices [15,16,17,18,19,20,21,22].

We hypothesized that, under identical solvent conditions, HC would provide enhanced recovery of bioactive compounds compared with UAE and conventional solid–liquid extraction due to differences in cavitation regime and mass transfer dynamics. To quantitatively assess extraction performance, the primary endpoints were defined a priori as the recovery of key flavanone markers (eriocitrin and hesperidin), total phenolic content, and antioxidant capacity evaluated through complementary DPPH and ORAC assays.

Therefore, the aim of this work was to establish a solvent-optimized framework for the comparative evaluation of UAE and HC for phenolic recovery from lemon processing by-products. By integrating solvent optimization, advanced extraction technologies, and post-extraction concentration, this study proposes a reproducible and scalable approach for enhancing the recovery of bioactive compounds from citrus processing side-streams and provides new insights into the performance of HC as an emerging technology for sustainable phenolic extraction.

## 2. Materials and Methods

### 2.1. Chemicals and Reagents

The following reagents were employed in this study: Folin–Ciocalteu reagent (FCR), sodium carbonate (Na_2_CO_3_), gallic acid, fluorescein, 2,2′-azobis (2-amidinopropane) dihydrochloride (AAPH), 6-hydroxy-2,5,7,8-tetramethylchroman-2-carboxylic acid (Trolox), and 2,2-diphenyl-1-picrylhydrazyl (DPPH), all obtained from Sigma-Aldrich (St. Louis, MO, USA) and of analytical grade. Phenolic standards (eriocitrin, hesperidin, caffeic acid, *p*-coumaric acid, sinapic acid, and ferulic acid) used for chromatographic identification and quantification were obtained from Sigma-Aldrich (St. Louis, MO, USA). Solvents used for chromatographic analyses were of HPLC grade (Merck KGaA, Darmstadt, Germany).

### 2.2. Plant Material

Lemon fruits (*Citrus limon* (L.) Burm. f.) were harvested at commercial maturity, corresponding to fully developed fruits with typical external coloration for the species. The fruits were obtained from experimental orchards located at the Palazzelli site (Siracusa, Italy), belonging to the Council for Agricultural Research and Economics (CREA), Research Center for Olive, Fruit and Citrus Crops (Acireale, Italy). Post-processing by-products (commonly referred to as “pastazzo”), consisting of peel, pulp, and seeds, were obtained immediately after juice extraction using a laboratory-scale mechanical citrus juicer. To ensure sample representativeness, lemon fruits were randomly collected from multiple trees across the orchard, and a total of 300 fruits were processed. The resulting lemon by-products consisted mainly of peel fragments, pulp, and seeds. Fresh by-products were immediately homogenized using a blender (Simac, Bravosimac FS 300, Lugo, Italy) and processed promptly to preserve their bioactive composition. The homogenized material was dehydrated in a ventilated oven at 50–55 °C (Memmert, UM 500, Schwabach, Germany) until the moisture content was reduced below 5%, with drying times typically ranging between 24 and 48 h depending on sample thickness and airflow conditions, allowing safe storage and preventing microbial degradation prior to extraction. The dried material was then manually fragmented and further milled using a mechanical grinder (Moulinex, Mx Type A505, Ecully Cedex, France) to obtain a coarse and homogeneous particle size (5–10 mm fragments). For all extraction experiments, the dried lemon by-products were used at a solid-to-solvent ratio of 1:10 (*w*/*v*). Water–ethanol mixtures (0–100% *v*/*v* ethanol) were initially evaluated to identify the optimal solvent system for phenolic recovery prior to the comparative extraction experiments.

### 2.3. Solvent Optimization by Conventional Solid–Liquid Extraction

A series of conventional laboratory-scale extractions was performed to identify the optimal solvent composition for phenolic recovery from dried lemon by-products. For each extraction, 100 g of dried lemon by-products were mixed with 1.0 L of solvent (solid-to-solvent ratio 1:10 *w*/*v*) and subjected to continuous magnetic stirring at 200 rpm for 60 min using a magnetic stirrer (VELP Scientifica, Type AGE, Usmate Velate, Italy). During extraction, temperature was monitored using a calibrated digital thermometer (TFA Dostmann, 30.2018.02, Wertheim, Germany) and maintained below 50 °C in order to preserve thermolabile compounds and ensure process stability. Extractions were performed using water–ethanol mixtures with increasing ethanol proportions, ranging from 0% to 100% ethanol (*v*/*v*) in 10% increments. After extraction, the liquid phase was separated by filtration to remove residual solids. The obtained extracts were subsequently analyzed for phenolic content and antioxidant activity. Among the tested solvent systems, the 50:50 (*v*/*v*) water–ethanol mixture showed the highest extraction efficiency and was therefore selected as the optimal solvent system for subsequent experiments. The selected hydroalcoholic extract was subjected to rotary evaporation under reduced pressure using a rotary evaporator (Rotavapor RE-111) equipped with a water bath (B-461), both from Büchi (Flawil, Switzerland), at a controlled temperature (below 50 °C) to remove ethanol and concentrate the solution, yielding a stable aqueous extract. This optimized solvent system was then used to standardize the subsequent comparative evaluation of UAE and HC.

### 2.4. Ultrasound-Assisted Extraction (UAE)

UAE was performed using a bath sonicator (model C.E.E.M.30A, Sarl R.E.U.S., Drap, France) operating at a frequency of 24 kHz with a maximum nominal power of 1.25 kW. A mechanical overhead stirrer was used simultaneously to maintain constant agitation at 200 rpm during the extraction process. For each extraction, 1 kg of dried lemon by-products was mixed with 10 L of a 50:50 (*v*/*v*) water–ethanol solution in a glass extraction vessel. The vessel was placed centrally inside the ultrasonic bath according to the manufacturer’s operational recommendations. Bath loading conditions were kept constant for all experiments by maintaining identical bath liquid levels, vessel geometry, and working volume in order to ensure consistent acoustic energy transmission. Sonication was carried out in six consecutive cycles of 10 min each (total extraction time: 60 min), with short pauses between cycles to limit temperature increase. Temperature was continuously monitored using a calibrated digital thermometer and maintained below 50 °C to preserve thermolabile compounds. Based on the nominal ultrasonic power (1.25 kW) and the working extraction volume (10 L), the ultrasound power density was approximately 125 W/L. Although acoustic energy distribution in ultrasonic baths is inherently heterogeneous, this parameter provides a useful reference for experimental reproducibility and comparison with other UAE systems. At the end of extraction, the suspension was filtered to remove solid residues, and the resulting filtrate was collected for subsequent concentration and analytical determination. Ethanol was removed under reduced pressure using a rotary evaporator under the same conditions described in Section 2.3 to obtain a concentrated aqueous extract suitable for comparison with conventional extraction and HC extracts.

### 2.5. Hydrodynamic Cavitation (HC) Extraction

HC extraction was performed using a centrifugal cavitator system (model Tiny (T) Lab Unit Soldo, Three-ES S.r.l., Lazzate, Italy) coupled with a volumetric pump (model SFA90CA4, Liverani S.r.l., Lugo, Italy) with a maximum flow capacity of 0.55 m^3^/h. For the experimental setup, the flow rate was regulated at 0.92 L/min (0.055 m^3^/h), a condition previously identified as optimal for balancing cavitation intensity and thermal stability [16]. The extraction process was carried out using 1.5 kg of dried lemon by-products suspended in 15 L of a 50:50 (*v*/*v*) water–ethanol solution. The cavitation treatment consisted of six consecutive cycles of 10 min each, for a total extraction time of 60 min. Temperature was continuously monitored using a calibrated digital thermometer and maintained below 50 °C to prevent degradation of thermolabile compounds. The cavitation device was operated at a motor rotational frequency of 60 Hz, which has been reported to promote stable cavitation conditions and efficient mass transfer in similar extraction systems [16,17]. Based on the operating flow rate, pressure conditions at the inlet and outlet of the cavitation device, and the reactor geometry, the extraction process was conducted under stable cavitation conditions with an estimated cavitation number below unity (σ < 1). Operating under these hydrodynamic conditions promotes the formation of intense cavitation phenomena, generating localized shear forces, microturbulence, and enhanced solvent–matrix interactions that facilitate the release of phenolic compounds from plant tissues. After completion of the cavitation treatment, the suspension was filtered to remove solid residues. The obtained hydroalcoholic extract was subsequently concentrated under reduced pressure by rotary evaporation under the same conditions described in Section 2.3, yielding a stable aqueous phase for subsequent chemical analyses and direct comparison with conventional extraction and UAE.

### 2.6. HPLC–PDA Analysis of Phenolic Compounds

Phenolic compounds in lemon extracts were identified and quantified using high-performance liquid chromatography coupled with a photodiode array detector (HPLC–PDA), following previously described procedures with minor modifications [5]. Chromatographic separation was performed on a reversed-phase Luna C18 column (250 × 4.6 mm, 5 μm; Phenomenex, Torrance, CA, USA) using a Surveyor HPLC system (Thermo Electron Corporation, San Jose, CA, USA) equipped with a Surveyor photodiode array (PDA) detector and controlled by ChromQuest software (version 3.1.6, Thermo Electron Corporation, San Jose, CA, USA). Prior to analysis, samples were filtered through a 0.45 μm membrane filter (Albet, Barcelona, Spain). The mobile phase consisted of water containing 0.3% formic acid (solvent A) and acetonitrile containing 0.3% formic acid (solvent B). The gradient program was as follows: 0 min, 5% B; 10 min, 20% B; 50 min, 28% B; 60 min, 43% B; followed by an isocratic step at 43% B for 10 min and subsequent re-equilibration of the column to initial conditions. The flow rate was set at 1 mL/min, the column temperature was maintained at 30 °C, and the injection volume was 20 μL. Identification of phenolic compounds was achieved by comparing retention times and UV spectra with those of authentic analytical standards. Quantification was performed using external calibration curves for each compound. Flavanones (eriocitrin and hesperidin) were monitored and quantified at 280 nm, whereas hydroxycinnamic acids (caffeic, *p*-coumaric, sinapic, and ferulic acids) were monitored and quantified at 310 nm. These compounds were selected as target phenolic markers for evaluating extraction performance. All analyses were performed in triplicate analytical determinations for each extract. Concentrations of individual phenolic compounds were expressed as mg/L of the corresponding standard compound. Results were expressed as mg/L of extract to allow a direct comparison of extraction performance among the different extraction techniques and processing conditions investigated.

The HPLC–PDA analytical method was validated in terms of linearity, sensitivity, and accuracy (Table 1). External calibration curves were constructed for eriocitrin, hesperidin, caffeic acid, *p*-coumaric acid, sinapic acid, and ferulic acid over compound-specific concentration ranges covering the expected levels in the analyzed samples. Calibration curves showed excellent linearity with coefficients of determination (R^2^) equal to 0.999 for all analytes. Limits of detection (LOD) and quantification (LOQ) were estimated using signal-to-noise criteria. LOD values ranged from 0.03 to 1.67 mg/L, while LOQ values ranged from 0.10 to 5.00 mg/L depending on the compound. Method accuracy was evaluated through recovery experiments performed by spiking representative samples within the linear range of each analyte. Recovery values ranged from 98.6% to 99.9%, confirming the suitability of the analytical method for quantitative determination of phenolic compounds in lemon by-product extracts.

### 2.7. Determination of Total Polyphenol Content

The total polyphenol content of lemon by-product extracts was determined using a colorimetric assay based on the Folin–Ciocalteu method, following previously established protocols with slight modifications [5]. In brief, 1.0 mL of each filtered extract was diluted in 10 mL of ultrapure water. An aliquot of 1 mL from this dilution was then combined with 5 mL of a 10% (*v*/*v*) FCR and 4 mL of a 7.5% (*w*/*v*) Na_2_CO_3_ solution. The resulting mixture was incubated for 2 h at ambient temperature in the absence of light. Absorbance was subsequently measured at 765 nm using a Varian Cary 100 Scan UV-Vis spectrophotometer (Varian, Palo Alto, CA, USA). All measurements were performed in three analytical replicates for each extract, and total polyphenols were expressed as mg gallic acid equivalents (GAE)/L of extract.

### 2.8. Oxygen Radical Absorbance Capacity (ORAC) Assay

The antioxidant capacity of the lemon by-product extracts was assessed using the ORAC assay, following previously described protocols with minor adjustments [23,24]. Fluorescence measurements were performed using a Wallac 1420 Victor III microplate reader (PerkinElmer, Waltham, MA, USA) equipped with excitation and emission filters set at 485 nm and 535 nm, respectively. Fluorescein (116 nM) served as the fluorescent probe, while AAPH (153 mM) was employed as a source of peroxyl radicals. The reaction mixture was incubated at 37 °C in a 75 mM phosphate buffer (pH 7.0). Trolox was used as the reference antioxidant standard, and phosphate buffer was included as the blank. All solutions were freshly prepared on the day of analysis. Prior to measurement, samples were diluted in phosphate buffer at an appropriate ratio (1:25, *v*/*v*) to ensure that fluorescence decay curves fell within the assay’s linear range. ORAC values were expressed as micromoles of Trolox equivalents (µmol TE) per 100 mL of extract. All measurements were performed in three analytical replicates.

### 2.9. DPPH Radical Scavenging Activity Assay

The radical scavenging activity of lemon extracts was assessed using the DPPH assay, following methodological adaptations from previously published protocols [25,26]. A 0.6 mM DPPH solution was freshly prepared in absolute ethanol. For each analysis, 200 µL of lemon extract was added to 2.0 mL of the DPPH solution and mixed thoroughly. The reaction mixtures were incubated in the dark at room temperature for 30 min to allow the reaction to proceed. The decrease in absorbance was measured at 517 nm using a Varian Cary 100 Scan UV-Vis spectrophotometer (Varian, Palo Alto, CA, USA). Antioxidant activity was expressed as the percentage of DPPH radical inhibition, calculated according to the following equation:

DPPH scavenging activity (%) = [(A_blank_ − A_sample_)/A_blank_] × 100

where A_blank_ represents the absorbance of the control solution (DPPH in ethanol without extract), and A_sample_ represents the absorbance of the reaction mixture containing the extract. All measurements were performed in three analytical replicates.

### 2.10. Statistical Analysis

All experimental data, including individual phenolic compounds determined by HPLC, total polyphenols, and antioxidant activity (ORAC and DPPH assays), were subjected to statistical evaluation using Statgraphics Plus software, version 4.1 (Manugistics Inc., Rockville, MD, USA). One-way analysis of variance (ANOVA) was applied to determine significant differences among extraction treatments. When ANOVA indicated statistical significance (*p* < 0.05), mean comparisons were carried out using Duncan’s multiple range test. Statistical differences are indicated by different letters in tables and figures. Results were expressed as mean ± standard deviation (SD) of triplicate analytical determinations.

## 3. Results and Discussion

### 3.1. HPLC Identification of Phenolic Compounds

**Figure 1:**
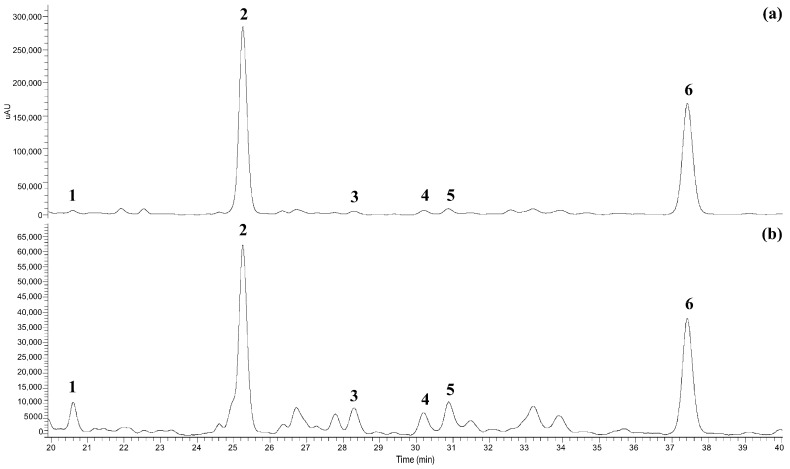
HPLC chromatographic profiles of phenolic compounds in lemon by-product extracts recorded at 280 nm (**a**) and 310 nm (**b**): (1) caffeic acid; (2) eriocitrin; (3) *p*-coumaric acid; (4) sinapic acid; (5) ferulic acid; (6) hesperidin.

A preliminary qualitative HPLC–PDA analysis was performed to identify the main phenolic compounds present in lemon by-product extracts obtained using the optimized solvent system. Representative chromatographic profiles are shown in Figure 1. The chromatograms revealed the presence of six major phenolic compounds belonging to two principal classes: flavanones and hydroxycinnamic acids. Compound identification was achieved by comparing retention times and UV spectra with those of authentic analytical standards. Chromatographic detection was performed at two wavelengths: 280 nm for flavanones (eriocitrin and hesperidin) and 310 nm for hydroxycinnamic acids (caffeic, *p*-coumaric, sinapic, and ferulic acids). These compounds were selected as target phenolic markers for subsequent quantitative comparison among the different extraction treatments.

Eriocitrin and hesperidin were the most abundant flavanones detected and are widely recognized as characteristic phenolic constituents of *Citrus limon* matrices [27,28]. Eriocitrin is predominantly associated with lemon peel and juice and has been reported to exhibit strong antioxidant activity and good stability during processing [29], while hesperidin, although more abundant in sweet orange, is also present in lemon matrices and contributes to the functional properties of citrus extracts [30]. Within the hydroxycinnamic acid group, caffeic, *p*-coumaric, sinapic, and ferulic acids were consistently detected across samples. These compounds are commonly reported in citrus processing by-products and may occur in both free and matrix-associated forms, contributing to the overall antioxidant capacity of the extracts and potentially interacting synergistically with flavonoids [31].

The phenolic profile identified in this study is consistent with previous reports on lemon by-products and citrus matrices [32,33], supporting the reliability of the analytical approach. The predominance of flavanones over hydroxycinnamic acids is also in agreement with quantitative trends reported for lemon-derived matrices. The identification of these phenolic markers provided the analytical basis for the subsequent quantitative evaluation of extraction efficiency among the different extraction strategies investigated. Quantitative results are presented and discussed in the following sections.

### 3.2. Effect of Solvent Composition on Extraction Efficiency and Antioxidant Activity

**Figure 2:**
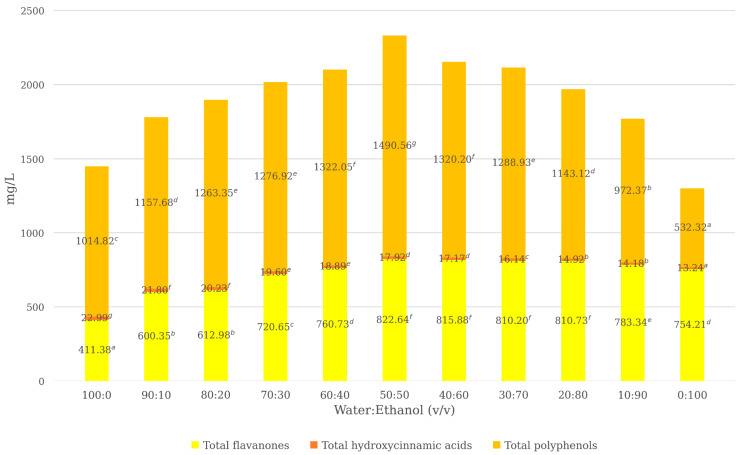
Effect of water–ethanol ratio (*v*/*v*) on phenolic compounds and total polyphenols in lemon by-product extracts obtained by conventional lab-scale extraction. Total flavanones were calculated as the sum of eriocitrin and hesperidin, while total hydroxycinnamic acids were calculated as the sum of caffeic, *p*-coumaric, sinapic, and ferulic acids. Phenolic compounds are expressed as mg/L of the corresponding standard compound, whereas total polyphenols are expressed as mg gallic acid equivalents (GAE)/L of extract. Values represent the mean of triplicate analytical determinations. Different superscript letters indicate statistically significant differences among water–ethanol ratios according to one-way ANOVA followed by Duncan’s multiple range test (*p* < 0.05).

The influence of the water–ethanol ratio (*v*/*v*) on phenolic recovery and antioxidant capacity was investigated using conventional laboratory-scale extraction under controlled conditions. As reported in Table 2 and illustrated in Figure 2, solvent composition had a pronounced effect on both individual phenolic compounds and total phenolic fractions.

As shown in Table 2, eriocitrin exhibited increasing extraction efficiency with rising ethanol content up to 50%, followed by a progressive decline at higher ethanol proportions. In contrast, hesperidin showed a continuous increase across the entire range of ethanol concentrations, which may be related to its lower aqueous solubility and greater affinity for ethanol-rich solvent systems. Hydroxycinnamic acids (caffeic, ferulic, *p*-coumaric, and sinapic acids) displayed an opposite trend, generally decreasing with increasing ethanol content, indicating that higher ethanol concentrations reduce the extraction efficiency of more polar phenolic compounds. These results highlight the influence of solvent polarity on the extraction behavior of different phenolic compounds and are consistent with previous studies reporting that binary solvent systems improve plant matrix permeability and enhance phenolic release through cell wall disruption [7,34]. In particular, water promotes matrix swelling and facilitates mass transfer, whereas ethanol enhances the solubilization of less polar phenolics [35], resulting in different extraction trends depending on compound polarity.

Among the tested conditions, the 50:50 (*v*/*v*) water–ethanol mixture provided the highest recovery of total flavanones and total polyphenols, reaching 822.64 mg/L and 1490.56 mg GAE/L, respectively (Figure 2), while hydroxycinnamic acids showed higher values at lower ethanol proportions, as observed in both Table 2 and Figure 2. Although individual compounds exhibited different optimal conditions, the 50:50 mixture ensured the best overall balance in the recovery of bioactive compounds. This behavior is in line with previous studies reporting that hydroalcoholic mixtures with intermediate ethanol content provide optimal conditions for phenolic recovery from citrus by-products [7,34], as they ensure an effective balance between solvent polarity and compound solubility, enabling the simultaneous extraction of compounds with different polarity characteristics.

The antioxidant capacity of the extracts obtained with different solvent compositions was evaluated using two complementary assays: the ORAC assay, based on a hydrogen atom transfer (HAT) mechanism, and the DPPH radical scavenging assay, which relies on an electron transfer (ET) mechanism. The combined use of these assays allows a more comprehensive characterization of antioxidant activity, as they reflect distinct radical neutralization pathways [36]. In addition, the Folin–Ciocalteu method, commonly used to determine total polyphenol content, was considered an indicator of antioxidant potential, as previously reported for citrus and other plant-derived matrices [16,37,38].

As shown in Table 3, solvent composition significantly influenced the antioxidant capacity of the extracts, producing trends that generally mirrored phenolic extraction efficiency, although with assay-dependent differences. The DPPH radical scavenging activity exhibited a clear positive correlation with total polyphenol content, increasing from 15.56% in the aqueous extract to a maximum of 25.70% in the 50:50 (*v*/*v*) water–ethanol extract, followed by a decrease at higher ethanol concentrations. This increase is consistent with literature reports describing a strong dependence of DPPH activity on phenolic concentration in citrus-derived extracts [39,40]. The observed trend reflects the sensitivity of the DPPH assay to phenolic hydroxyl groups and its strong association with the redox potential of polyphenolic structures.

Conversely, ORAC values showed an opposite trend with respect to ethanol concentration, with the highest antioxidant capacity observed in the aqueous extract (10,978.41 µmol TE/100 mL) and progressively decreasing as ethanol content increased, reaching a minimum value in the 100% ethanol extract (6049.34 µmol TE/100 mL). A similar inverse relationship between ORAC values and increasing ethanol content has been reported for citrus matrices, where water-rich solvents favor the extraction of hydrophilic antioxidants contributing to HAT-based activity [39,41]. This trend can be attributed to the preferential extraction of hydrophilic antioxidants in water-rich solvents, including polar phenolic acids and other water-soluble reducing compounds naturally present in lemon by-products [39].

Overall, these results highlight that the 50:50 (*v*/*v*) water–ethanol mixture represents the most balanced solvent system, maximizing phenolic recovery and DPPH radical scavenging activity while maintaining substantial ORAC. This observation is in agreement with previous studies indicating that solvent composition modulates not only total phenolic yield but also the relative contribution of compounds involved in ET- and HAT-based antioxidant mechanisms [34,36]. Antioxidant capacity is influenced not only by the quantity of extracted phenolics but also by their qualitative composition under different solvent conditions. Statistical analysis confirmed significant differences among solvent systems for all evaluated parameters (*p* < 0.05). Therefore, this hydroalcoholic mixture was selected as the optimal solvent system for the subsequent comparative evaluation of UAE and HC.

### 3.3. Comparative Evaluation of Conventional Extraction, UAE, and HC for Phenolic Recovery and Antioxidant Activity

Following solvent optimization, the 50:50 (*v*/*v*) water–ethanol mixture was identified as the most effective solvent system for recovering phenolic compounds from lemon by-products. This result is consistent with literature reporting that hydroalcoholic mixtures provide an optimal balance between solvent polarity and compound solubility for phenolic extraction from plant matrices [34]. In particular, water facilitates the extraction of highly polar compounds, while ethanol enhances the recovery of moderately polar phenolics, resulting in a synergistic extraction effect. Moreover, ethanol–water systems are widely recognized as food-grade, low-toxicity solvents suitable for industrial applications. It should be noted that alternative green solvents, such as NaDES and glycerol-based systems, have also been proposed for phenolic extraction [12,34], particularly in the context of citrus by-product valorization [3]. However, their effectiveness strongly depends on solvent composition and target compounds, and a direct comparison was beyond the scope of the present study. Therefore, while the selected hydroalcoholic system provided optimal performance under the tested conditions, it may not be universally optimal for all phenolic classes, highlighting the need for further investigation. This solvent system was therefore used as the reference condition for the comparative evaluation of extraction methods: conventional laboratory-scale extraction, UAE, and HC. All methods were tested under identical extraction conditions, including extraction time, temperature, and solid-to-solvent ratio, to ensure a rigorous comparison of extraction efficiency. To further assess the influence of post-processing, both the hydroalcoholic extracts and their concentrated counterparts obtained after ethanol removal were analyzed in terms of phenolic composition and antioxidant activity.

As reported in Table 4, both UAE and HC significantly outperformed the conventional extraction method in the recovery of all major phenolic compounds. These findings are consistent with previous studies reporting the effectiveness of UAE in enhancing phenolic recovery from citrus by-products, primarily due to cavitation-induced cell disruption and improved mass transfer mechanisms [13,14]. Similarly, HC has been shown to intensify extraction processes through the generation of localized high shear forces and microturbulence, facilitating the release of intracellular compounds [15,16,17,18,19,20,21].

Eriocitrin increased from 664.83 mg/L in the conventional extract to 764.63 mg/L with UAE (+15%) and further to 864.82 mg/L with HC (+13% compared with UAE), confirming a progressive enhancement in extraction efficiency. A similar trend was observed for hesperidin, which increased from 157.81 mg/L to 271.35 mg/L with UAE (+72%) and to 295.52 mg/L with HC (+8.9% compared with UAE). Hydroxycinnamic acids exhibited comparable improvements, with caffeic acid increasing by 12.3% with UAE and by an additional 22.9% with HC, while *p*-coumaric acid increased by 19.7% and 12.5%, respectively. Sinapic acid and ferulic acid showed smaller but consistent increases, further supporting the overall trend of enhanced recovery under cavitation-based conditions, in agreement with literature data reporting improved phenolic extraction in citrus matrices under comparable conditions [13,14,15,16,17,18,19,20,21].

The observed differences among individual phenolic compounds may be attributed to their distinct chemical structures and interactions within the plant matrix, which influence their susceptibility to cavitation-induced release [19,20,21]. These results confirm the enhanced capacity of cavitation-based techniques to disrupt plant cellular structures and promote the release of both free and matrix-associated compounds.

After ethanol removal under reduced pressure, all extracts showed a marked increase in phenolic concentration, although the magnitude of this improvement depended strongly on the extraction method (Table 4). Such concentration effects are well-documented in the literature, where solvent removal has been shown to increase the apparent concentration of phenolic compounds and improve extract stability [34,42].

The concentrated conventional extract reached 994.62 mg/L of eriocitrin and 256.47 mg/L of hesperidin. In comparison, the UAE-concentrated extract exhibited 1094.72 mg/L (+10.1% vs. conventional) and 473.14 mg/L (+84.5% vs. conventional), while the HC-concentrated extract achieved the highest values, reaching 1349.85 mg/L (+23.3% vs. UAE) and 502.78 mg/L (+6.3% vs. UAE) for eriocitrin and hesperidin, respectively. Similar trends were observed for hydroxycinnamic acids. Compared with the concentrated conventional extract, UAE increased recovery by 5.7%, 12.9%, 15.44%, and 22.1% for caffeic, *p*-coumaric, sinapic, and ferulic acids, respectively, while HC further improved yields over UAE by 11.4%, 13.0%, 9.0%, and 13.7% for the same compounds. These concentration-dependent increases are in agreement with previous reports describing the combined effect of extraction and solvent removal in enhancing the apparent phenolic content of plant-derived extracts [34,42].

These findings highlight the synergistic effect between intensified extraction techniques and post-extraction concentration steps, further confirming the effectiveness of combining advanced extraction technologies with concentration processes to enhance phenolic recovery.

**Figure 3:**
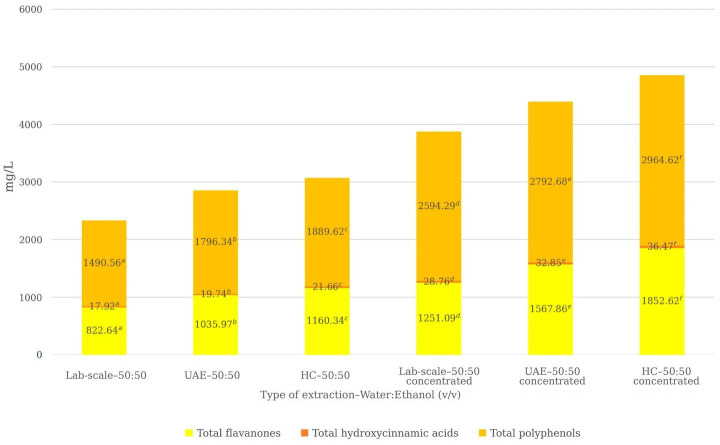
Effect of extraction technology on phenolic compounds and total polyphenols in lemon by-product extracts obtained using a 50:50 (*v*/*v*) water–ethanol system, including both non-concentrated and concentrated extracts. Total flavanones were calculated as the sum of eriocitrin and hesperidin, while total hydroxycinnamic acids were calculated as the sum of caffeic, *p*-coumaric, sinapic, and ferulic acids. Phenolic compounds are expressed as mg/L of the corresponding standard compound, and total polyphenols are expressed as mg gallic acid equivalents (GAE)/L of extract. Values represent the mean of triplicate analytical determinations. Different superscript letters indicate statistically significant differences among extraction methods according to one-way ANOVA followed by Duncan’s multiple range test (*p* < 0.05).

Considering cumulative phenolic classes (Figure 3), both UAE and HC significantly improved extraction performance relative to the conventional method. In non-concentrated extracts, UAE increased total flavanones by 25.9%, total hydroxycinnamic acids by 10.3%, and total polyphenols by 20.5%. HC provided additional improvements over UAE of 12.0%, 7.2%, and 5.2%, respectively. These increases are consistent with literature data reporting enhanced recovery of total phenolics in citrus matrices treated with cavitation-based techniques [15,16,17,18,19,20,21]. After concentration, these differences became even more pronounced. UAE-concentrated extracts showed increases of 25.3% (total flavanones), 17.5% (total hydroxycinnamic acids), and 7.7% (total polyphenols) compared with the concentrated conventional extract, while HC-concentrated extracts exhibited the highest recoveries overall, with further increases of 18.1%, 7.7%, and 6.2%, respectively, relative to UAE.

These results confirm that the combined effect of cavitation-based extraction and solvent removal enhances overall phenolic recovery beyond that achieved by each process individually.

As illustrated in Figure 3, extraction efficiency increased progressively from conventional extraction to UAE and HC, with HC consistently providing the highest recoveries, particularly after the concentration step. This progressive increase is in line with literature demonstrating that intensified extraction techniques enhance the recovery of bioactive compounds from agro-industrial by-products by promoting mass transfer and cell disruption phenomena [34,42]. While previous studies have reported the benefits of ultrasound- and microwave-assisted extraction in citrus matrices, the application of HC to lemon by-products remains largely unexplored.

This study therefore represents one of the first comprehensive evaluations of HC applied specifically to lemon processing by-products, providing quantitative evidence of its superior extraction efficiency compared with UAE and conventional extraction methods.

The higher extraction efficiency observed for HC can be explained by fundamental differences in cavitation generation and energy distribution between the two technologies. In UAE, cavitation is produced by acoustic waves, generating localized and often heterogeneous cavitation zones within the extraction medium. This phenomenon has been widely reported in ultrasound-based extraction systems, where uneven distribution of acoustic energy may lead to non-uniform cavitation intensity within the medium [13,14]. Although effective at enhancing mass transfer, this spatial heterogeneity may limit solvent–matrix interactions and reduce the uniformity of cavitational effects, particularly at larger volumes. In contrast, HC generates cavitation through controlled pressure variations induced by fluid acceleration, producing a more homogeneous cavitation field throughout the system. Such hydrodynamic conditions enable a more uniform energy distribution and improved contact between solvent and plant matrix, as reported for continuous-flow cavitation systems [15,16,17,18,19,20,21].

The collapse of cavitation bubbles in HC produces high-intensity microjets, shockwaves, and strong shear forces that promote extensive disruption of plant cell walls and facilitate the release of both free and bound phenolic compounds. These effects have been extensively described as key mechanisms responsible for enhanced extraction efficiency in cavitation-based processes [15,16,17,18,19,20,21], leading to improved solvent penetration into the plant matrix and accelerated mass transfer, resulting in higher extraction yields compared with UAE under similar operating conditions.

In addition, the continuous-flow configuration of HC allows repeated and uniform exposure of the solid–liquid suspension to cavitation phenomena, improving process uniformity and scalability compared with the predominantly batch-based operation of UAE. The scalability and process intensification potential of HC systems have been increasingly recognized in recent studies focusing on sustainable extraction technologies [22,43]. Combined with its operational simplicity and relatively short processing times, these characteristics make HC particularly attractive for industrial applications within circular bioeconomy frameworks [43].

The particularly high concentrations of eriocitrin and hesperidin detected in HC extracts further confirm their role as characteristic flavanone markers of lemon matrices, consistent with previous compositional studies of *Citrus limon* by-products [7,44]. Likewise, the enrichment of hydroxycinnamic acids, including caffeic, *p*-coumaric, sinapic, and ferulic acids contributes to the antioxidant potential of the recovered fractions and supports their potential use in nutraceutical and cosmetic applications.

Such compositional profiles further confirm the relevance of citrus-derived phenolic compounds as natural antioxidants for food, nutraceutical, and cosmetic formulations [4,5,44]. Overall, these results demonstrate that process integration, combining optimized solvent systems, advanced extraction technologies, and post-extraction concentration, is an effective strategy for maximizing bioactive recovery from citrus processing by-products.

As shown in Table 5, the antioxidant activity of lemon by-product extracts was significantly influenced by both extraction technology and post-extraction concentration. Across all evaluated parameters, including total polyphenols, ORAC, and DPPH scavenging activity, the application of advanced extraction technologies (UAE and HC) resulted in clear improvements compared with the conventional lab-scale extraction method.

These results are consistent with previous studies reporting that intensified extraction processes enhance the recovery and functionality of antioxidant compounds from plant matrices by improving mass transfer and compound accessibility [13,45].

In non-concentrated extracts, UAE increased total polyphenols by +20.5%, ORAC by +9.6%, and DPPH activity by +6.0% relative to the conventional extraction. This increase is consistent with previous studies demonstrating that UAE enhances antioxidant recovery through cavitation-induced effects that promote compound release and improve matrix disruption, facilitating the extraction of both free and matrix-associated antioxidants [13,45].

The transition from UAE to HC led to further improvements, with increases of +5.2% for total polyphenols, +2.0% for ORAC, and a more pronounced +11.4% for DPPH activity. These additional gains are in agreement with recent studies highlighting the higher cavitation intensity and process efficiency of hydrodynamic systems compared with acoustic cavitation [15,16,17,18,19,20,21].

The stronger increase observed for DPPH suggests that HC may be particularly effective in extracting phenolic compounds with high redox potential, likely due to the more intense shear forces and microturbulence associated with HC phenomena [43].

In concentrated extracts obtained after ethanol removal, all antioxidant parameters increased markedly, confirming a strong synergistic effect between extraction technology and post-extraction concentration. The concentrated conventional extract already exhibited a substantial increase compared with its non-concentrated counterpart. However, the highest antioxidant values were observed in extracts obtained after UAE and HC followed by concentration. Compared with the concentrated conventional extract, UAE-concentrated samples showed increases of +7.6% for total polyphenols, +29.4% for ORAC, and +13.0% for DPPH activity. HC-concentrated extracts exhibited the highest antioxidant performance overall, with additional increases of +6.2% for total polyphenols, +3.7% for ORAC, and +4.6% for DPPH relative to UAE-concentrated extracts. These results confirm the key role of post-extraction concentration in enhancing antioxidant activity, in agreement with previous findings on polyphenol-rich extracts from agro-industrial by-products [34,42].

The pronounced enhancement of ORAC values observed after UAE and HC suggests that these technologies may favor the preservation or extraction of hydrogen atom transfer (HAT)-reactive antioxidants, including water-soluble peptides, organic acids, and low-molecular-weight polyphenols [46,47]. In contrast, the close correspondence observed between DPPH activity and total polyphenol content confirms that phenolic compounds remain strong predictors of electron transfer (ET)-based antioxidant capacity in citrus-derived extracts [48,49]. This complementary behavior between HAT- and ET-based assays highlights the importance of using multiple analytical approaches to comprehensively evaluate antioxidant potential [46,47,48,49].

Overall, these findings demonstrate that integrating solvent optimization, green extraction technologies, and post-extraction concentration enhances the antioxidant potential of lemon by-products. Among the evaluated techniques, HC consistently delivered the highest performance, indicating its potential as an efficient and scalable strategy for the valorization of citrus processing by-products, in line with recent studies emphasizing the relevance of efficient extraction technologies within circular bioeconomy frameworks [22,43].

### 3.4. Industrial and Functional Implications

The findings of this study provide practical insights and demonstrate potential industrial relevance for the sustainable valorization of lemon by-products, in line with the principles of green chemistry and the circular bioeconomy. The recovery of high-value compounds from agro-industrial side-streams has been widely explored in recent studies [6,7]. A key strength of this investigation lies in the standardized comparison of extraction methods, as all experimental variables, including temperature, extraction time, solid-to-solvent ratio, and solvent composition (50:50 *v*/*v* water:ethanol), were deliberately kept constant. This experimental alignment enabled a rigorous and direct comparison of extraction efficiency, focusing on bioactive yield and antioxidant potential while minimizing the influence of confounding factors.

Under these controlled conditions, both UAE and HC significantly enhanced the recovery of total flavanones, total hydroxycinnamic acids, and total polyphenols, with HC consistently outperforming both UAE and the conventional extraction method in terms of yield and antioxidant activity. The magnitude of these improvements, observed under identical processing conditions, highlights the effectiveness of cavitation-based technologies in enhancing compound recovery and improving the functional properties of the resulting extracts. These findings are consistent with previous studies reporting the effectiveness of ultrasound and other intensified extraction techniques in improving the recovery of phenolic compounds from plant matrices [13,34,42].

From an industrial perspective, the cost–benefit profile of HC appears favorable. Conventional extraction and UAE are typically implemented as batch processes, although UAE can also be applied in continuous-flow systems using probe-type configurations, which are widely adopted at the industrial scale due to their improved energy efficiency and process control. In contrast, HC operates in continuous-flow mode, enabling easier scalability, reduced handling times, and potentially reduced operational costs during extended processing periods. The advantages of continuous-flow cavitation systems in terms of scalability and process intensification have been increasingly reported in the recent literature on green extraction technologies [22,43]. Furthermore, HC can be readily integrated with solvent recovery systems and downstream concentration steps, which may further improve both economic efficiency and environmental sustainability.

In addition to these technological advantages, HC promotes efficient solvent–matrix interactions, extensive disruption of plant cellular structures, and enhanced extraction of both free and bound phenolic compounds. These mechanisms are well-documented for cavitation-based processes and are considered key factors contributing to improved extraction performance [15,16,17,18,19,20,21]. The combination of high extraction efficiency, operational simplicity, and continuous processing capability suggests that HC represents a promising and scalable technology for industrial implementation, particularly in the food, nutraceutical, and cosmetic sectors, where the demand for natural antioxidants and functional ingredients is steadily increasing.

It should be noted that, although HC inherently offers advantages related to process intensification and continuous-flow operation, key industrial parameters such as specific energy consumption, process throughput, and solvent recovery efficiency were not directly evaluated in the present study. Energy consumption represents a critical factor in the selection of extraction technologies, as the most efficient process in terms of yield is not necessarily the most economically sustainable. Although a detailed quantitative assessment of energy consumption was beyond the scope of this study, the nominal operating conditions of the investigated systems suggest qualitative differences in energy distribution and process efficiency. In particular, UAE operates at relatively high nominal power levels, whereas HC relies on fluid dynamic conditions to generate cavitation phenomena, potentially enabling improved energy utilization at larger processing scales. These considerations highlight the need for comprehensive techno-economic and life cycle assessments when comparing extraction technologies for industrial applications. Similar limitations have been reported in previous studies, emphasizing the importance of such approaches to fully validate the industrial sustainability of emerging extraction technologies [22,43].

Overall, this study proposes a replicable, scalable, and sustainable extraction strategy integrating solvent optimization, advanced cavitation-based extraction technologies, and post-processing concentration steps. Such an approach may also be extended beyond citrus by-products to other agro-industrial side-streams, contributing to waste valorization, resource circularity, and the advancement of circular bioeconomy models, as highlighted in the recent literature on sustainable food processing and by-product valorization [6,7,22].

## 4. Conclusions

This study demonstrates that solvent optimization, combined with a standardized comparison of extraction technologies, is an effective strategy for the recovery of antioxidant compounds from lemon processing by-products. The 50:50 (*v*/*v*) water:ethanol solvent system provided the best overall balance for phenolic extraction, enabling a direct comparison among conventional extraction, UAE, and HC under identical conditions. HC consistently achieved the highest yields of bioactive compounds and exhibited superior antioxidant activity, especially after ethanol removal and extract concentration.

These results highlight the key role of solvent composition, extraction technology, and post-extraction treatment in maximizing the recovery of bioactive compounds from citrus by-products in a sustainable and resource-efficient manner.

From an industrial perspective, the continuous-flow configuration of HC, together with its relatively short processing times and compatibility with food-grade solvents, suggests that this technology may be suitable for integration into existing citrus-processing chains. The results therefore indicate potential for the valorization of citrus processing by-products at the industrial scale, promoting their conversion into high-value ingredients for food, nutraceutical, and cosmetic applications.

The integration of solvent optimization with a standardized and controlled comparison of extraction technologies provides a robust framework for assessing emerging extraction processes and further supports the superior efficiency of HC for phenolic recovery from citrus processing by-products.

It should be emphasized that the present conclusions are based on comparative extraction performance evaluated under controlled experimental conditions rather than on a full techno-economic or energy balance assessment. Although the results clearly demonstrate the technological potential of HC, the experimental activities were conducted at the pilot scale, and a comprehensive techno-economic or life cycle assessment was not performed. Future research should therefore focus on techno-economic evaluation, life cycle analyses, and scale-up validation to fully support industrial implementation.

## Figures and Tables

**Table 1:** Validation parameters of the HPLC-PDA method used for the quantitative determination of flavanones and hydroxycinnamic acids in lemon by-product extracts.

Validation Parameters	Eriocitrin	Hesperidin	Caffeic Acid	*p*-Coumaric Acid	Sinapic Acid	Ferulic Acid
Calibration range (mg/L)	5.001–250.0	1.428–357.0	0.101–15.4	0.102–15.9	0.103–17.6	1.030–25.7
Equation	y = 262921x	y = 252167x	y = 977234x	y = 1474936x	y = 656519x	y = 646428x
R^2^	0.999	0.999	0.999	0.999	0.999	0.999
LOD (mg/L)	1.67	0.48	0.03	0.03	0.03	0.34
LOQ (mg/L)	5.00	1.43	0.10	0.10	0.10	1.03
Recovery (%)	98.8	98.6	99.9	99.4	99.9	98.9

**Table 2:** Concentration of phenolic compounds in lemon by-product extracts obtained by conventional lab-scale extraction using different water–ethanol ratios.

Water:Ethanol (*v*/*v*)	Eriocitrin	Hesperidin	Caffeic Acid	*p*-Coumaric Acid	Sinapic Acid	Ferulic Acid
100:0	337.39 ^a^ ± 4.23	73.99 ^a^ ± 0.37	4.66 ^c^ ± 0.01	4.88 ^f^ ± 0.11	9.64 ^f^ ± 0.02	3.81 ^g^ ± 0.07
90:10	505.62 ^b^ ± 3.35	94.73 ^b^ ± 0.88	4.51 ^c^ ± 0.01	4.33 ^e^ ± 0.14	9.41 ^f^ ± 0.25	3.55 ^f^ ± 0.03
80:20	516.33 ^b^ ± 5.11	96.66 ^b^ ± 0.52	4.31 ^c^ ± 0.01	3.60 ^d^ ± 0.02	8.95 ^e^ ± 0.04	3.37 ^e^ ± 0.01
70:30	595.21 ^e^ ± 2.30	125.43 ^c^ ± 0.54	3.74 ^b^ ± 0.01	3.58 ^d^ ± 0.10	8.94 ^e^ ± 0.01	3.33 ^d^ ± 0.03
60:40	609.46 ^f^ ± 0.71	151.27 ^d^ ± 1.92	3.75 ^b^ ± 0.03	3.08 ^c^ ± 0.03	8.83 ^e^ ± 0.08	3.24 ^d^ ± 0.04
50:50	664.83 ^i^ ± 1.17	157.81 ^e^ ± 1.13	3.50 ^a^ ± 0.05	2.95 ^c^ ± 0.05	8.63 ^d^ ± 0.02	2.84 ^c^ ± 0.04
40:60	648.51 ^h^ ± 2.08	167.38 ^f^ ± 1.41	3.41 ^a^ ± 0.02	2.76 ^b^ ± 0.02	8.34 ^d^ ± 0.05	2.66 ^b^ ± 0.01
30:70	624.48 ^g^ ± 2.59	185.72 ^g^ ± 3.97	3.32 ^a^ ± 0.02	2.58 ^b^ ± 0.02	7.62 ^c^ ± 0.12	2.62 ^b^ ± 0.02
20:80	618.33 ^f^ ± 1.61	192.40 ^h^ ± 0.98	3.24 ^a^ ± 0.01	2.38 ^a^ ± 0.01	6.74 ^b^ ± 0.04	2.56 ^b^ ± 0.01
10:90	584.26 ^d^ ± 1.16	199.08 ^i^ ± 1.12	3.59 ^a^ ± 0.40	2.25 ^a^ ± 0.02	5.82 ^a^ ± 0.01	2.52 ^a^ ± 0.01
0:100	554.71 ^c^ ± 2.26	199.50 ^i^ ± 0.47	3.17 ^a^ ± 0.05	2.17 ^a^ ± 0.02	5.47 ^a^ ± 0.07	2.43 ^a^ ± 0.01

Data are expressed as mg/L of the corresponding standard compound. Values represent the mean ± standard deviation (SD) of triplicate analytical determinations. Different superscript letters within the same column indicate statistically significant differences according to one-way ANOVA followed by Duncan’s multiple range test (*p* < 0.05).

**Table 3:** Antioxidant-related parameters of lemon by-product extracts obtained by conventional lab-scale extraction using different water–ethanol ratios (*v*/*v*).

Water:Ethanol (*v*/*v*)	Total Polyphenols (mg GAE/L)	ORAC (µmol TE/100 mL)	DPPH Scavenging Activity (%)
100:0	1014.82 ^c^ ± 4.29	10,978.41 ^j^ ± 19.72	15.56 ^a^ ± 0.09
90:10	1157.68 ^d^ ± 3.28	10,684.84 ^i^ ± 6.58	15.77 ^a^ ± 0.03
80:20	1263.35 ^e^ ± 3.22	10,456.92 ^h^ ± 14.46	15.83 ^a^ ± 0.01
70:30	1276.92 ^e^ ± 3.72	10,017.88 ^g^ ± 17.27	16.67 ^b^ ± 0.08
60:40	1322.05 ^f^ ± 23.61	9854.14 ^f^ ± 21.79	21.50 ^f^ ± 0.18
50:50	1490.56 ^g^ ± 7.54	9721.33 ^e^ ± 19.98	25.70 ^i^ ± 0.02
40:60	1320.20 ^f^ ± 2.05	8683.74 ^d^ ± 11.33	25.25 ^h^ ± 0.03
30:70	1288.93 ^e^ ± 4.29	7776.36 ^c^ ± 23.93	24.39 ^g^ ± 0.02
20:80	1143.12 ^d^ ± 1.65	6519.97 ^b^ ± 16.48	19.18 ^e^ ± 0.08
10:90	972.37 ^b^ ± 3.22	6093.49 ^a^ ± 3.73	18.40 ^d^ ± 0.14
0:100	532.32 ^a^ ± 0.25	6049.34 ^a^ ± 4.72	17.65 ^c^ ± 0.07

Values represent the mean ± standard deviation (SD) of triplicate analytical determinations. Different superscript letters within the same column indicate statistically significant differences according to one-way ANOVA followed by Duncan’s multiple range test (*p* < 0.05).

**Table 4:** Concentration of individual phenolic compounds in lemon by-product extracts obtained using different extraction technologies (conventional lab-scale extraction, UAE, and HC) under optimized solvent conditions.

Type of Extraction	Water:Ethanol (*v*/*v*)	Eriocitrin	Hesperidin	Caffeic Acid	*p*-Coumaric Acid	Sinapic Acid	Ferulic Acid
Lab-scale	50:50	664.83 ^a^ ± 1.17	157.81 ^a^ ± 1.13	3.50 ^a^ ± 0.05	2.95 ^a^ ± 0.05	8.63 ^a^ ± 0.02	2.84 ^a^ ± 0.04
UAE	50:50	764.63 ^b^ ± 0.74	271.35 ^c^ ± 0.57	3.93 ^b^ ± 0.07	3.53 ^b^ ± 0.02	9.34 ^b^ ± 0.04	2.94 ^a^ ± 0.05
HC	50:50	864.82 ^c^ ± 1.04	295.52 ^d^ ± 0.72	4.83 ^c^ ± 0.01	3.97 ^b^ ± 0.02	9.83 ^c^ ± 0.01	3.03 ^b^ ± 0.01
Lab-scale	50:50 concentrated	994.62 ^d^ ± 5.72	256.47 ^b^ ± 0.29	5.48 ^d^ ± 0.08	5.24 ^c^ ± 0.31	13.33 ^d^ ± 0.04	4.71 ^c^ ± 0.07
UAE	50:50 concentrated	1094.72 ^e^ ± 5.28	473.14 ^e^ ± 2.39	5.79 ^e^ ± 0.01	5.92 ^d^ ± 0.01	15.39 ^e^ ± 0.07	5.75 ^d^ ± 0.02
HC	50:50 concentrated	1349.85 ^f^ ± 0.47	502.78 ^f^ ± 2.09	6.45 ^f^ ± 0.05	6.69 ^e^ ± 0.01	16.78 ^f^ ± 0.02	6.54 ^e^ ± 0.04

Phenolic compounds are expressed as mg/L of the corresponding standard compound. Values represent the mean ± standard deviation (SD) of triplicate analytical determinations. “Concentrated” samples refer to extracts obtained after ethanol distillation. Different superscript letters within the same column indicate statistically significant differences according to one-way ANOVA followed by Duncan’s multiple range test (*p* < 0.05).

**Table 5:** Antioxidant-related parameters of lemon by-product extracts obtained using different extraction technologies (conventional lab-scale extraction, UAE, and HC) under optimized solvent conditions.

Type of Extraction	Water:Ethanol (*v*/*v*)	Total Polyphenols (mg GAE/L)	ORAC (µmol TE/100 mL)	DPPH Scavenging Activity (%)
Lab-scale	50:50	1490.56 ^a^ ± 7.54	9721.33 ^a^ ± 19.98	25.70 ^a^ ± 0.02
UAE	50:50	1796.34 ^b^ ± 1.39	10,656.77 ^b^ ± 19.48	27.23 ^b^ ± 0.08
HC	50:50	1889.62 ^c^ ± 6.47	10,874.14 ^c^ ± 27.50	30.33 ^c^ ± 0.06
Lab-scale	50:50 concentrated	2594.29 ^d^ ± 3.28	12,478.20 ^d^ ± 10.99	59.52 ^d^ ± 0.04
UAE	50:50 concentrated	2792.68 ^e^ ± 7.07	16,141.98 ^e^ ± 30.32	67.23 ^e^ ± 0.08
HC	50:50 concentrated	2964.62 ^f^ ± 9.31	16,732.97 ^f^ ± 22.92	70.33 ^f^ ± 0.06

Values represent the mean ± standard deviation (SD) of triplicate analytical determinations. “Concentrated” samples refer to extracts obtained after ethanol distillation. Different superscript letters within the same column indicate statistically significant differences according to one-way ANOVA followed by Duncan’s multiple range test (*p* < 0.05).

## Data Availability

All the data generated by this research have been included in the article. For any assistance, it is possible to contact the corresponding author.

## Abbreviations

The following abbreviations are used in this manuscript:

AAPH	2,2-Azobis(2-amidinopropane) Dihydrochloride
ANOVA	Analysis of Variance
DPPH	2,2-Diphenyl-1-picrylhydrazyl
ET	Electron Transfer
FCR	Folin–Ciocalteu Reagent
GAE	Gallic Acid Equivalents
HAT	Hydrogen Atom Transfer
HC	Hydrodynamic Cavitation
HPLC	High-Performance Liquid Chromatography
HPLC-PDA	High-Performance Liquid Chromatography–Photodiode Array Detector
LOD	Limit of Detection
LOQ	Limit of Quantification
Na_2_CO_3_	Sodium Carbonate
NaDES	Natural Deep Eutectic Solvents
ORAC	Oxygen Radical Absorbance Capacity
SD	Standard Deviation
TE	Trolox Equivalents
Trolox	6-hydroxy-2,5,7,8-tetramethylchroman-2-carboxylic acid
UAE	Ultrasound-Assisted Extraction
UV-Vis	Ultraviolet–Visible Spectroscopy
